# Construction of Quantitative Structure Activity Relationship (QSAR) Models to Predict Potency of Structurally Diversed Janus Kinase 2 Inhibitors

**DOI:** 10.3390/molecules24234393

**Published:** 2019-12-01

**Authors:** Saw Simeon, Nathjanan Jongkon

**Affiliations:** 1 Thailand Center of Excellence for Life Sciences (Public Organization), Ministry of Science and Technology, Bangkok 10400, Thailand; saw.s@ku.th; 2 Department of Social and Applied Science, College of Industrial Technology, King Mongkut’s University of Technology North Bangkok, Bangkok 10800, Thailand

**Keywords:** tyrosine kinase inhibitors, quantitative structure activity relationship, data mining, Janus kinase 2

## Abstract

Janus kinase 2 (JAK2) inhibitors represent a promising therapeutic class of anticancer agents against many myeloproliferative disorders. Bioactivity data on pIC${}_{50}$ of 2229 JAK2 inhibitors were employed in the construction of quantitative structure-activity relationship (QSAR) models. The models were built from 100 data splits using decision tree (DT), support vector machine (SVM), deep neural network (DNN) and random forest (RF). The predictive power of RF models were assessed via 10-fold cross validation, which afforded excellent predictive performance with $R^{2}$ and RMSE of 0.74 ± 0.05 and 0.63 ± 0.05, respectively. Moreover, test set has excellent performance of $R^{2}$ (0.75 ± 0.03) and RMSE (0.62 ± 0.04). In addition, Y-scrambling was utilized to evaluate the possibility of chance correlation of the predictive model. A thorough analysis of the substructure fingerprint count was conducted to provide insights on the inhibitory properties of JAK2 inhibitors. Molecular cluster analysis revealed that pyrazine scaffolds have nanomolar potency against JAK2.

## 1. Introduction

Cancer exerts a great impact on the quality of life and is a leading cause of death worldwide. Although cancer chemotherapy, one of the major medical advances in the last few decades, is directed toward certain macromolecules to treat cancer, it cannot efficiently discriminate between normally dividing cell and tumor cells, leading to unwanted toxic side effects. However, targets are usually located in tumor cells, thus providing a high specificity toward tumor cells and broader therapeutic window with less toxicity is beneficial. Therefore, targeted therapy represents a promising approach to cancer therapy [1]. Generally an ideal therapeutic target should not only be susceptible to specific inhibition by small ligands but tumor cells also more dependent on the activity of the target than normal cells [2].

Janus kinase 2 (JAK2) is a member of the Janus family of tyrosine kinase, which plays an important role in many cellular signaling pathways [3,4]. It is a nonreceptor tyrosine kinase that relays signals from cytokine receptors to downstream targets, including the transcription factors STAT3 and STAT5. When it is activated, this family of enzymes increase tumor cell proliferation and growth, induce antiapoptotic effects and promote angiogenesis as well as metastasis [5,6]. Therefore, the inhibition of JAK2 would greatly reduce the activity of tyrosine kinase and compounds achieving such effects are known as JAK2 inhibitors [7].

JAK inhibitors are important class of targeted therapy that interfere with specific cell signaling pathways, which allows target-specific therapy for selected malignancies. Many of the JAK inhibitors are known to interfere with the JAK-STAT pathways, which have an implication in the treatment of different types of cancers and inflammatory diseases [8,9,10,11]. JAK inhibitors can be found in FDA approved drugs and clinical trials. For example, Ruxolitinib, an orally bioavailable selective inhibitor of JAK2, inhibits the proliferation of JAK2 [12]. Lestautinib, an orally bio-available polyaromatic indolocarbozole alkaloid, is a tyrosine kinase inhibitor that is currently in clinical trials and assigned Investigational New Drug (IND) number 76431 [13].

Quantitative structure activity relationship (QSAR) is an approach for elucidating the origin of biological activity with their respective chemical compounds represented as descriptors. The QSAR models can reveal molecular features that are essential for active compounds and that can subsequently be used as therapeutic agents [14]. Several QSAR models were developed in the hope to drive the novel compounds with better properties against kinase [15,16,17,18,19,20,21,22]. To understand the origin and bioactivities of JAK inhibitors, models were developed with the hope to identify important pharmacophores and substructures using pharmacophores and 3D QSAR [23,24,25,26,27,28,29,30,31,32,33,34,35]. Due to the polypharmacological nature of compounds, multi-target QSAR models have been also developed to handle the interaction of multiple targets of JAK inhibitors. Although pharmacophores and 3D QSAR models, as well as multi-target QSAR models [36,37] and tools [38] are essential in understanding structure-activity relationship of JAK2 inhibitors, the ability of the those models to predict unknown bioactivity properties depends largely on the size of training sets. Extrapolation power of the model, where the model predicts accurately with confidence and credibility, depends on how well the training data represent the unknown compounds. Therefore, QSAR model will have a small applicability domain and low general predictability if they are based on a small data set.

Here we propose a large-scale QSAR investigation for predicting JAK2 inhibitors. Several statistical methods were used to build regression models in which inhibitors were represented as highly interpretable substructure fingerprint descriptors to understand the underlying JAK2 inhibitory activity, which is performed according to the guidelines of Organisation for Economic Cooperation and Development (OECD) [39]. This may provide important insights into the structural basis for the inhibition of JAK2, which may aid in the fight against cancer, in particular myeloproliferative neoplasms.

## 2. Results

### 2.1. Chemical Space of JAK2 Inhibitors

**Figure 1:**
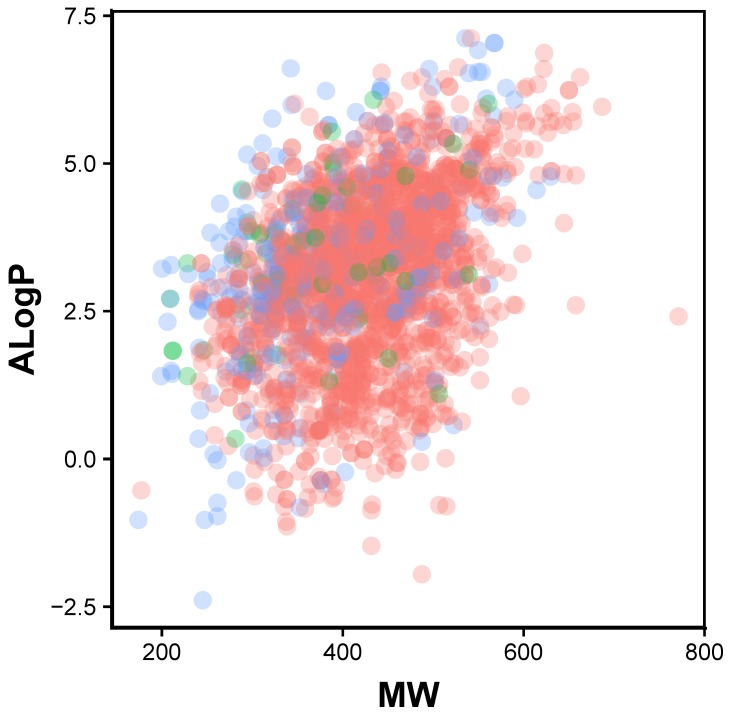
Chemical space of JAK2 inhibitors are shown as actives (green), inactives (red) and intermediates (blue).

**Figure 2:**
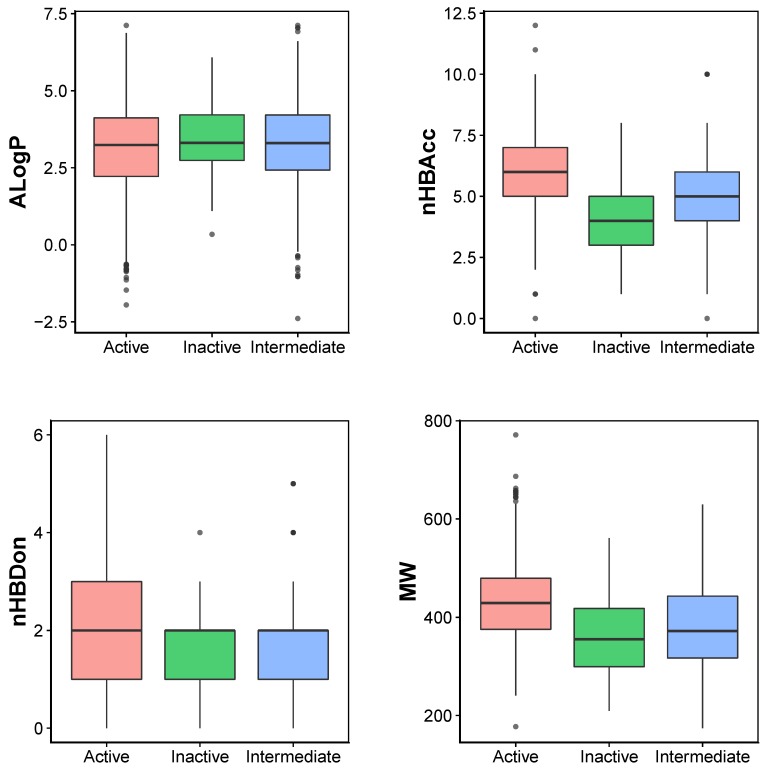
Box plot of the Linpiski’s descriptors actives (green), inactives (red) and intermediates (blue).

In order to provide the chemical space of JAK2 inhibitors, Lipinski’s rule-of-five descriptors are analyzed. This may provide insights on the origin of inhibitory properties of compounds. Lipinski’s rule-of-five descriptor consisted of molecular weight (MW), octanol-water partition coefficient (ALogP), number of hydrogen bond donors (nHBDon) and number of hydrogen bond acceptors (nHBAcc). Scatter plot of ALogP vs MW of the JAK2 inhibitors coloured by activities is shown in Figure 1. It can be seen that most of the compounds lie in the space of approximately 300 to 500 Da (MW) and 2.5 to 4 (AlogP). A boxplot of AlogP, nHBAcc, nHBDon and MW broken down by activity group is shown in Figure 2. Based on the boundaries of the boxes, there is no differences between the three bioactivity classes for nHBdon and ALogP. However, there is a weak trend of differences between the bioactivity groups for nHBAcc and MW, suggesting the active bioactivity classes higher nHBAcc and MW values. The results may suggest that the most desirable region for bioactivity is MW > 400, AlogP < 3 and nHBAcc > 6 (Figure 2).

### 2.2. QSAR Modeling

Usage of substructure fingerprint descriptors allows us to pinpoint the substructures that are important for modulating activity of JAK2. In order to get rid of the redundancy among the descriptors, the substructures were filtered using a cutoff threshold set at 0.70. As previously mentioned, the initial data set was split into a training set and test set, where the former represented 80% of the data set while the latter constituted the remaining 20% of the data set. To avoid the random seed, data splitting was performed for 100 iterations where each split was used to create a predictive model. The mean and standard deviation of the resulting predictive performance ($R^{2}$, RMSE, $r_{m}^{2}$ and $\Delta r_{m}^{2}$) were computed.

QSAR models were developed with various machine learning methods consisting of rule based models (DT), ensemble models (RF), non-linear models (SVM) and deep learning (DNN). As shown in the Table 1, the predictive performances of the training set provide $R^{2}$ of 0.65–0.75. The $R^{2}$ can be represented as intuitive metrics for ranking model and for intuitive comparison. The presence of irrelevant descriptors or overfitted model can be revealed by deterioration of predictive performance from 10-fold cross validation and a test set. A closer look at the models reveal that the bagging of trees improves the predictive performance over a single tree by reduction variance of the prediction. As seen in the Table 1, the $R^{2}$ of training set for RF is 0.75 ± 0.02 whereas for DT is 0.65 ± 0.02. SVM is an non-linear modeling technique which is considered to be powerful and highly flexible. The $R^{2}$, RMSE, $r_{m}^{2}$ and $\Delta r_{m}^{2}$ of the training set for SVM is 0.72 ± 0.02, 0.65 ± 0.02, 0.57 ± 0.04 and 0.26 ± 0.01, respectively. Recently, deep learning is an emerging technology in machine perception and natural language processing. The performance of DNN is higher than DT with $R^{2}$ of 0.59 ± 0.04 and RMSE = 0.82 ± 0.07. Table 2 showed the MAE of DT, SVM, DNN and RF. It can be seen that the order of error according to MAE is RF > SVM > DT > DNN for the training set. The error order is slightly different for the test set which is RF > SVM > DNN > DT. It can be seen that RF model is not overfitted to the training data which is indicated by the small gap between the training and test set MAE values. Several QSAR models on JAK2 were performed. The training set of 22, 31, 40, 42, 51 and 161 leads to the $R^{2}$ of 0.97 [28], 0.97 [23], 0.929 [27], 0.970 [26], 0.93 [25] and 0.869 [24], respectively. It can be seen that QSAR models built from lower training sets tend to have better performance. On the other hand, the QSAR built from a large data set using diverse chemical structures will have lower performance due to confounding factors. Nevertheless, QSAR models built from a large data set may have implication on the domain of applicability.

**Figure 3:**
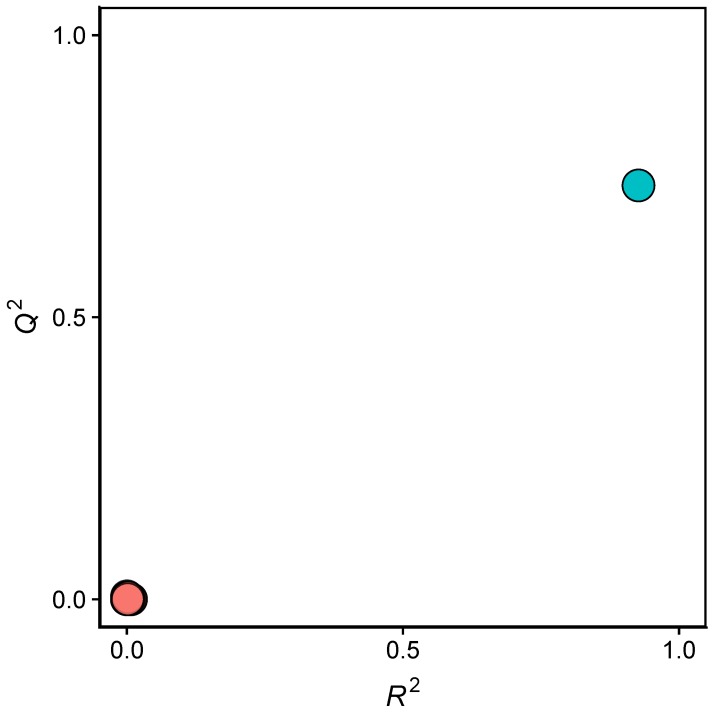
Y-scrambling plot of pIC${}_{50}$ as obtained from QSAR models after feature selection. The scrambled models in which the pIC${}_{50}$ were randomly shuffled while keeping the descriptor matrix intact. The scrambled models were coloured as pink while the real model was coloured as green.

Scatter plots of $R^{2}$ versus $Q^{2}$ for the Y-permutated (i.e., Y-scrambled) datasets of JAK2 inhibitory properties is shown in Figure 3. It can be observed that the actual X-Y pair for the QSAR models of bioactivities (pIC${}_{50}$) is clearly separated from the permutated X-Y pairs, ruling out the chance of correlation of the QSAR models [40].

**Figure 4:**
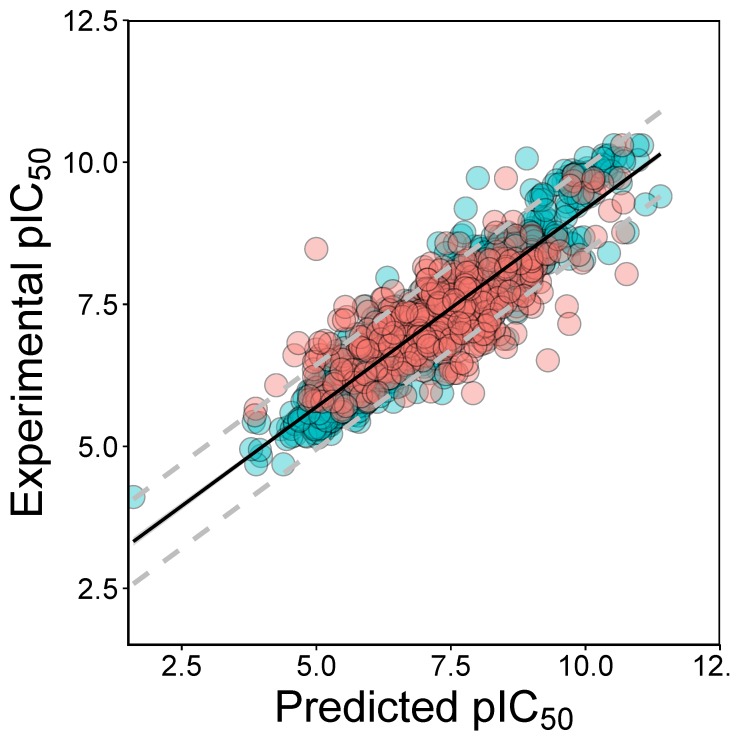
Experimental vs Predicted plot of pIC${}_{50}$ as obtained from QSAR models after feature selection. The training set and test set are shown as blue circles and red circles.

The models built on JAK2 has an excellent performance for RF as judged from the cross-validation set and test set. The performance of the cross-validation set is $R^{2}$ = 0.74 ± 0.05 and RMSE = 0.63 ± 0.05. For the test set, the RF have higher predictive performance as deduced from $R^{2}$ (0.75 ± 0.03) and RMSE (0.65 ± 0.04). The model complies with the requirement of the threshold values proposed by Tropsha ($R^{2}$ > 0.6 and $Q^{2}$ > 0.5) [41]. The margin between the $R^{2}$ of training set and $R^{2}$ of test set is 0.00, indicating that the model is reliable and predictive [42]. Figure 4 showed the experimental pIC${}_{50}$ as a function of prediction from RF.

### 2.3. Interpretation of QSAR Models

**Figure 5:**
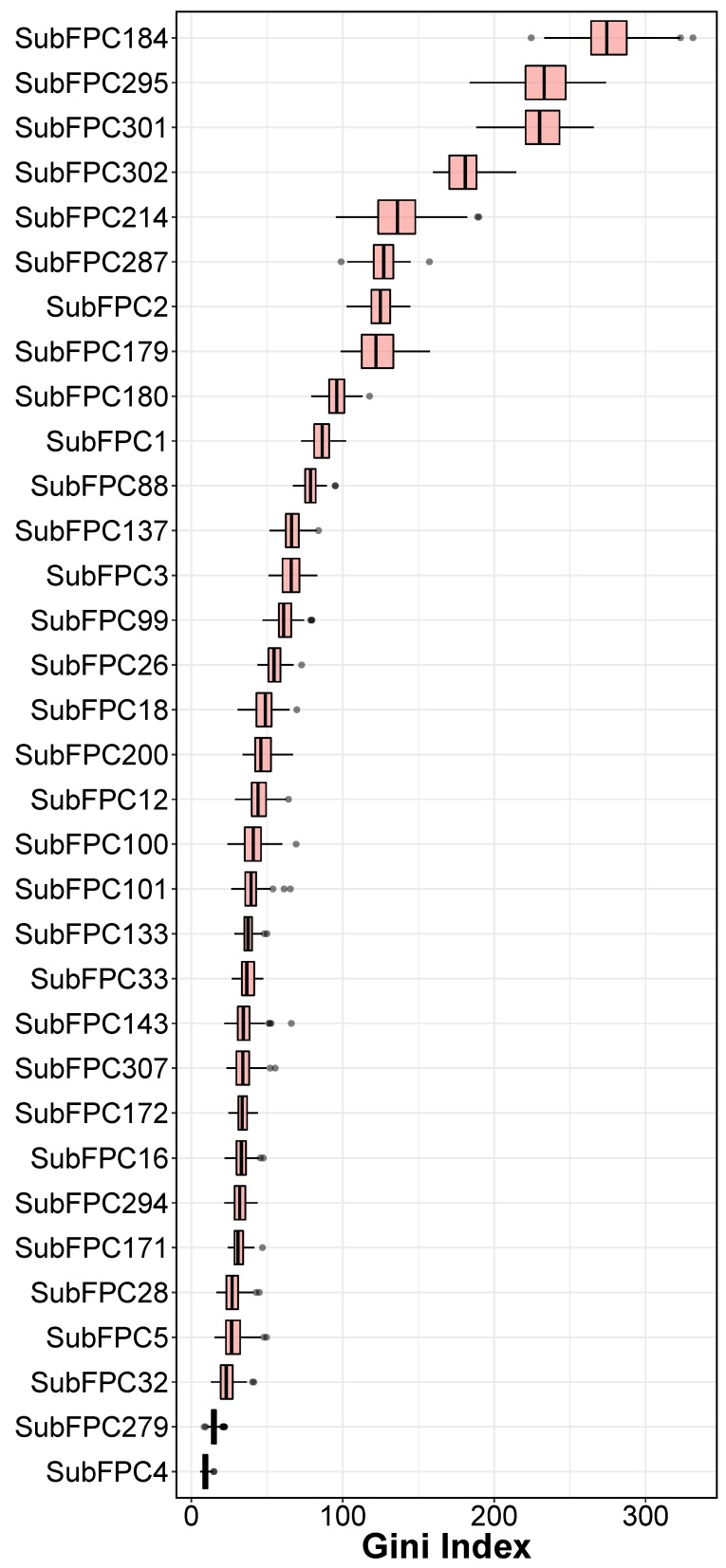
Gini Index of RF from selected descriptors.

The analysis of feature importance for different types of substructure fingerprints provides a better understanding of the JAK2 inhibitors. Table 3 showed a list of structure fingerprints and their descriptors that were utilized in the study. The efficient, effective and transparent Gini Index from RF was used to identity important features based on the predictive performance in Table 1. To avoid the bias of random seed in evaluating feature importance, the average and standard deviation values of Gini Index from 100 runs are used in the analysis. When interpreting the Gini Index, the high values have the most weight in dependent variables (pIC${}_{50}$). From the Figure 5, it can be seen that, SubFPC184 (276.48 ± 20.19), SubFPC295 (233.71 ± 17.86), SubFPC301 (230.87 ± 16.51), SubFPC302 (180.49 ± 11.78) and SubFPC214 (136.28 ± 19.00) have the highest values of Gini Index, suggesting that these substructures in compounds could have substantial impact on potency, based on the QSAR model. Because the features which have the highest coefficient values have highest weight of increase in bioactivity value, SubFPC184 (Heteroaromatic) is one of the most important features in determining potency of JAK2. It can be observed that FDA approved drugs namely Ruxolitinib [43], Tofacitinib [44], Baricitinib [45], Fedratinib [46], have heteroaromatic pyrimidine ring, suggesting that heteroaromatic is an important substituent when designing novel drugs as JAK2 inhibitors. The second most important feature is SubFPC295 which represents C ONS bond in the chemical structures. This feature facilitates in non-convalent interaction between inhibitors and JAK2 [47]. The third most important feature is SubFPC301 (1,5-Tautomerizable). Tautomerizable heterocycles have recently emerged as an attractive class of inhibitors for JAK2. Indeed, Pyrazolo[1,5-a]pyrimidines are important classes of chemical compounds that display a wide range of biological activities, including ant-cancer by modulating JAK2 [48]. Lastly, SUBFPC302 (rotatable bond) and SubFPC214 (sulfonic derivative) are important to consider when designing novel JAK2 inhibitors with high potency.The analysis of a crystal structure showed that selectivity of protein inhibitors is controlled by a hydrophobic pocket via a rotatable bond in the compound skeleton [49]. This is in agreement with the fact that all of the clinical approved drugs that target JAK2 has at least one rotatable bond in their chemical structures.

### 2.4. Applicability Domain

**Figure 6:**
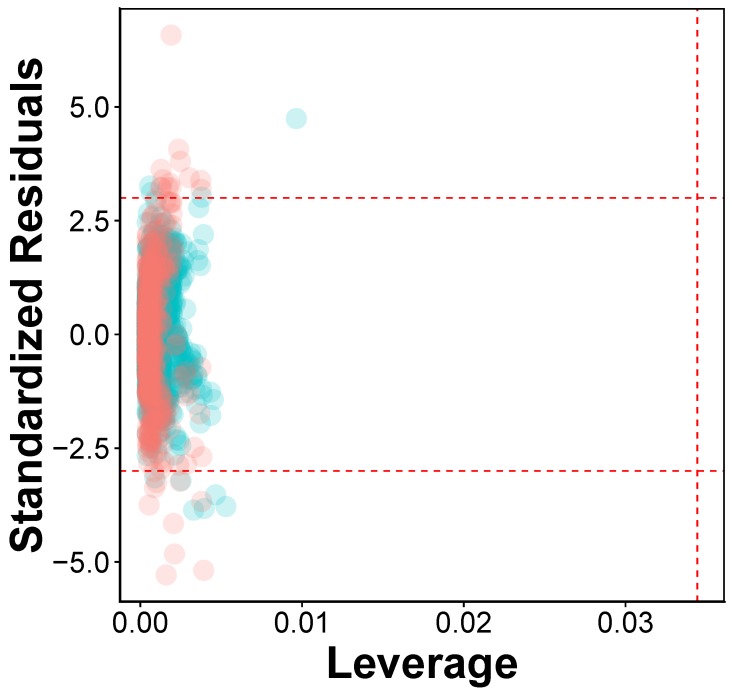
William plot for the QSAR model built using RF in which the horizontal dashed line represent ± 3 standardized residual and vertical dashed line represent warning leverage value (h*) of 0.034. The blue dots represent training set and the red dots represent test set.

The AD of the QSAR was defined as to assess the credibility of the model via the Williams plot, shown in Figure 6. The employed data set has in total 2229 compounds, which were partitioned into two separate subsets. The first subset consisted of 80% of the data set, which is used as training set while the second, set (20%) is used as a test set. Samples that represent the training set were highlighted as blue, whereas the test set was colored as red (Figure 6). The h* had a value of 0.034 for the QSAR model developed using RF. Clearly, it can be observed that almost all of the 2229 compounds are within the boundaries of applicability domain, indicating that the QSAR model had a well-defined AD. This may be because the training sample is based on various chemotypes, allowing the model to predict the test set with validity and credibility. On the other hand, there are a few compounds which lie outside the applicability domain of the model (Table S1).

### 2.5. Molecular Cluster Analysis of JAK2 Inhibitors

To identify privileged scaffolds, cheminformatics approach was utilized to deduce privileged scaffolds giving rise to high inhibitory activities against JAK2. Privileged substructures are a concept introduced in which they are capable of making compounds that display potency for more than one receptor, providing viable alternatives when searching for new receptor inhibitors [50].

**Figure 7:**
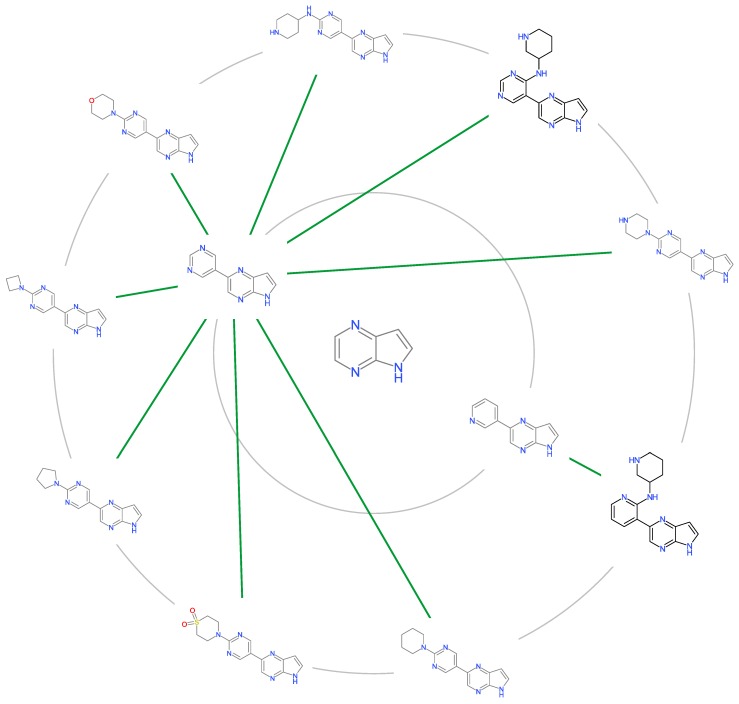
Scaffold Tree of Cluster 6 having nanomolar potency against JAK2.

Scaffolds analysis is performed with the following steps: (1) compounds are clustered within the Tanimoto Similarity of 0.80 (2) clusters N > 19 are retained for further analysis (3) mean pIC${}_{50}$ of each clusters are compared to the mean of JAK inhibitors (4) scaffolds are prioritized in terms of how much higher mean of the cluster when compared to mean of the dataset. Table 4 showed the mean pIC${}_{50}$ of each cluster in which cluster 5 and 6 have a nanomolar potency. A few exemplars can be purchased for each scaffolds for future screening in designing potent drugs candidates against JAK2 (Figure 7).

## 3. Materials and Methods

### 3.1. Data Set

**Figure 8:**
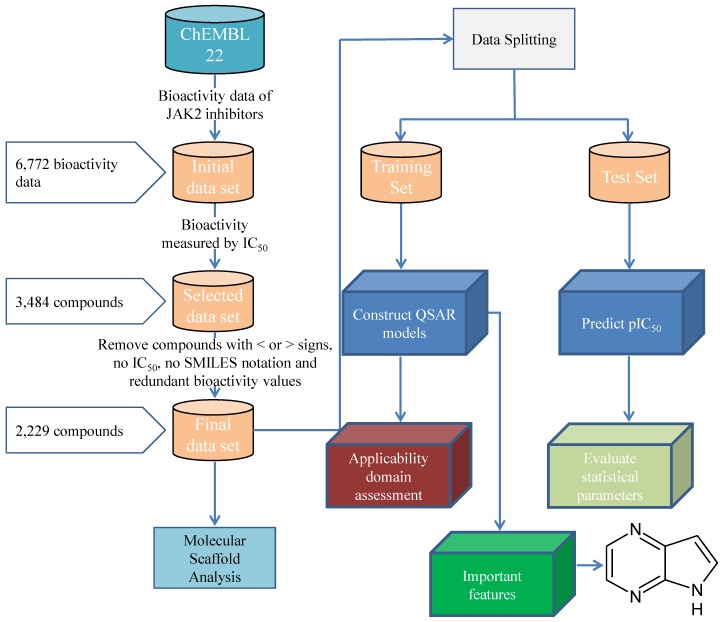
Workflow for the JAK2 QSAR Modelling.

A data set of inhibitors against the human JAK2 were compiled from the ChEMBL 22 database, which is comprised of a total number of 6772 bioactivity data points from 3906 compounds [51]. Compounds were treated with the QSAR curation workflow from Fourches et al. [52]. SMILES notations were treated with the ChemAxon’s Standardizer with the following options: Strip Salts, Aromatize, Clean 3D, Tautomerize, Neutralize, or Remove explicit hydrogens [53]. IC${}_{50}$ was selected for further investigation from the initial data set which possess several bioactivity measurement units (including IC${}_{50}$, Ki, % activity, % inhibition, MIC, EC50 etc) because it constitutes largest subset with 3484 compounds. Moreover, compounds with without reported IC${}_{50}$ values or having lesser/greater than signs were removed, resulting in 2229 compounds. The workflow for the JAK2 QSAR Modelling is shown in the Figure 8.

#### 3.1.1. Description of Compounds

Understanding biological, chemical and physical properties of chemical compounds is a central issue in pharmaceutical bioinformatics. With what accuracy this bioactivity can be predicted solely depends on how chemical compounds are described. Several molecular descriptors have been introduced with the aim of finding the most suited descriptors to relate these properties [54,55,56,57,58]. Here, substructure fingerprint [59] count was utilized to describe the JAK2 inhibitors using PaDEL-Descriptor software [60].

#### 3.1.2. Feature Selection

**Figure 9:**
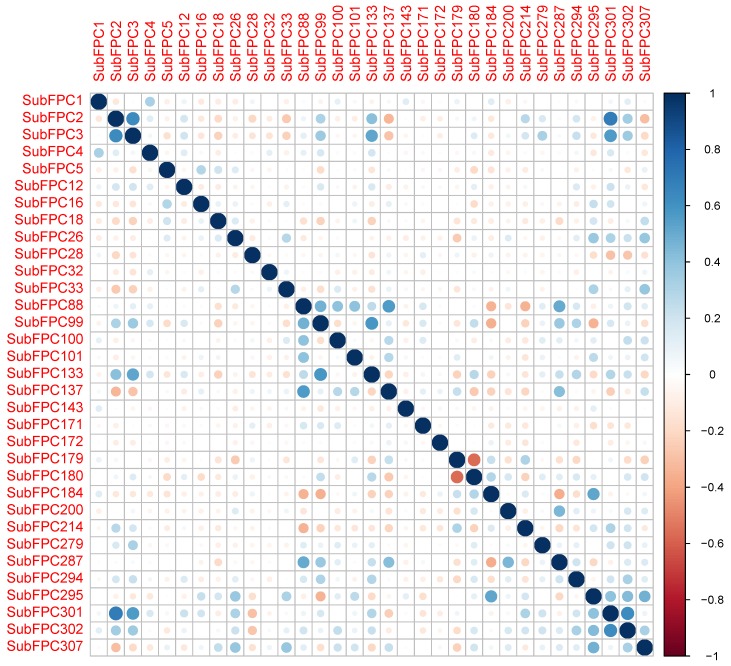
Intercorrelation matrix of the descriptors utilized for constructing the predictive models.

Collinearity is a condition where a pair of descriptors have a substantial correlation with each other. In general, it is desirable to avoid data with highly correlated predictors. Not only do redundant predictors frequently add more complexity to the model than the information they provide to the model, which adds computational cost and time, but they also over-fit the model [61,62]. Over-fitting means the model will usually have poor accuracy when predicting a new sample. Additionally, it also affects the interpretation of descriptors because the resulting coefficient estimates or feature usages are highly unstable [63]. In general, a Pearson’s correlation coefficient of 0.7 is an indicator of high collinearity among predictors [64]. Thus, *cor* function from the R package *stats* was used to calculate correlations among descriptors. To obtain filtered descriptors with all pairwise correlations less than 0.7, the *findCorrelation* function from the R package *caret* with a cutoff at 70% was used [65]. The remaining descriptors used in the study are shown in the Figure 9.

#### 3.1.3. Data Splitting

To avoid the bias that may arise from a single split when creating training models [66], predictive models were constructed from each of the 100 independent data splitting and the mean and standard deviation values of statistical parameters were reported. The dataset was randomly split (80%/20%) into training and independent test set. The *sample* function from the R *base* package was used to split the data [67]. Briefly, the sample function from R based is utilized to provide index numbers of rows for 80% as a training index from the whole data set. To obtain the training set, the training data index obtained from the sample function is utilized as index number to extract rows using brackets while the remaining rows (20%) were used as testing set.

#### 3.1.4. Multivariate Analysis

Supervised learning enables the model to make prediction about unseen or future data by learning from labeled training data. Regression models were constructed for the prediction of the continuous response variables as a function of predictors.

Decision Tree (DT) is a rule-based algorithm in which construction involves top steps, which are growing and pruning. Growing starts from root node which are branches out to form internal nodes. Internal nodes represent descriptors, branches describe descriptors value sand leaf nodes represent dependent variables (i.e., pIC${}_{50}$). The tree is reduced to a set of rules, which are eliminated via pruning for simplification. Advantage of pruning is that it reduces the complexity of the formed free and reduces the chance of over fitting. The *rpart* function from the R package *rpart* was used to build the QSAR models [68].

Support vector machine (SVM) is a machine learning that can be used to perform both classification and regression in which kernel function is used to map the data into high dimensional feature space. Commonly used guassian radial basis was used to build the model. The *svm* function from R package *e1071* was utilized to build QSAR models [69].

Deep neural network (DNN) is a method that imitate human brain comprising networks of interconnected neurons that function in relaying message in the form of electrochemical signals. DNN maps inputs to a target through a deep sequence of simple data transformations. The sequential model from R package *keras* was used to build QSAR models [70].

Random forest (RF) is an ensemble model that is comprised of multiple decision trees. Optimal tuning parameters (i.e., mtry) for RF were obtained by training the model with different ranges accompanied with 10-fold cross-validation. The *randomForest* function from the R package *randomForest* is used [71].

### 3.2. Validation of QSAR Models

Model validation is an essential process for assessing the performance of the predictive model. The following statistical metrics were used to evaluate the performance of the QSAR models: coefficient of determination ($R^{2}$) [72], root mean squared error (RMSE) [73], $r_{m}^{2}$ [74] and $\Delta r_{m}^{2}$ [74] as well as mean absolue error (MAE) [75]. The $R^{2}$ and RMSE are commonly utilized metric to assess the model performance. $r_{m}^{2}$ and $\Delta r_{m}^{2}$ metrics were used to verify the robustness of the proposed QSAR model where an acceptable QSAR model should give $r_{m}^{2}$> 0.5. Furthermore, 10-fold cross-validation, test set validation and Y-scrambling test were used to verify the predictive performance of the QSAR models.

The 10-fold cross-validation technique is one of the most frequent statistical evaluation in which 10 percent of data is left out as a test set while the remaining data is used to build model (*Q*${}^{2}$) [76]. This process is iterated until all the data has been left out as a test set. Y-scrambling test was also undertaken to assess the relationship between *R*${}^{2}$ and *Q*${}^{2}$ to rule of the possibility of chance correlation [40]. The original Y-dependent variable (i.e., pIC${}_{50}$) was randomly shuffled with respect to their associated independent variables (i.e., fingerprint descriptors).

### 3.3. Applicability Domain Analysis

The applicability domain is an essential concept in QSAR which can be used to estimate the uncertainty in prediction of a particular molecule based on the distance to the compounds used to build the model [77]. Leverage approach was utilized to identify whether a new compound will lie within or outside the domain, which was previously described [78]. The leverage is the distance between a molecule and the centroid of the space of training set. If a compound has standardized error of greater than 3 or less than -3 or higher than h${}^{*}$ then the prediction the compound is unreliable. The h${}^{*}$ can be computed using the following equation:

(1) $$h^{*}\;=\;\frac{3(p+1)}{n}$$

where *p* is the number of substructure fingerprint count and *n* is the number of samples in the training set.

### 3.4. Molecular Cluster Analysis

Binning clustering was utilized to cluster compounds in which the Tanimoto similarity cutoff is set at 0.8 to ensure that similar chemotypes are clustered in each group. The *cmp.cluster* function from the R package *ChemmineR* was employed to cluster the chemical structures [79]. Singletons were excluded as they do not provide information. The top cluster biased towards activity is annotated based on the Murcko Framework and displayed using the Scaffold Hunter [80].

## 4. Conclusions

Computational approaches for predicting the activities of JAK2 inhibitors can facilitate drug discovery efforts by saving cost and time. QSAR modeling was performed using the substructure fingerprint descriptors as an input to determine the importance on the inhibitory properties of the JAK2, which provided excellent predictive performance for both cross-validation and the test set. By utilizing the Gini Index of RF, heteroaromatic substituents (i.e. heteroaromatic ring, 1,5-tautomerizable hetrocyclis and rotatable bond) are shown to have significant weight in improving the potency of the JAK2 inhibitors. Molecular cluster analysis revealed that pyrazine scaffolds have nanomolar potency against JAK2. Such insights can provide a better understanding of the origin of the JAK2 inhibitory properties and may be used as a reference for designing novel modulators.

## Figures and Tables

**Table 1:** Performance summary of QSAR Models for predicting pIC${}_{50}$ using DT, SVM, DNN and RF.

Models	Training Set					10-Fold CV					Test Set			
	$R^{2}$	RMSE	$r_{m}^{2}$	$\Delta r_{m}^{2}$		$R^{2}$	RMSE	$r_{m}^{2}$	$\Delta r_{m}^{2}$		$R^{2}$	RMSE	$r_{m}^{2}$	$\Delta r_{m}^{2}$
DT	0.65 ± 0.02	0.72 ± 0.02	0.65 ± 0.02	0.28 ± 0.01		0.45 ± 0.07	0.91 ± 0.06	0.40 ± 0.09	0.20 ± 0.06		0.29 ± 0.05	1.02 ± 0.04	0.28 ± 0.05	0.31 ± 0.05
SVM	0.72 ± 0.01	0.65 ± 0.02	0.66 ± 0.02	0.26 ± 0.01		0.57 ± 0.05	0.80 ± 0.06	0.54 ± 0.04	0.33 ± 0.03		0.58 ± 0.05	0.79 ± 0.05	0.56 ± 0.03	0.33 ± 0.02
DNN	0.59 ± 0.04	0.82 ± 0.07	0.57 ± 0.04	0.32 ± 0.03		0.47 ± 0.07	0.93 ± 0.08	0.43 ± 0.07	0.29 ± 0.08		0.49 ± 0.04	0.90 ± 0.06	0.47 ± 0.04	0.31 ± 0.05
RF	0.75 ± 0.02	0.62 ± 0.02	0.69 ± 0.01	0.24 ± 0.01		0.74 ± 0.05	0.63 ± 0.05	0.67 ± 0.04	0.25 ± 0.03		0.75 ± 0.03	0.62 ± 0.04	0.68 ± 0.03	0.25 ± 0.02

**Table 2:** MAE of QSAR Models for DT, SVM, ANN and RF.

Models		Training Set		10-Fold CV		Test Set
		MAE		MAE		MAE
DT		0.53 ± 0.02		0.65 ± 0.05		0.76 ± 0.03
SVM		0.42 ± 0.02		0.55 ± 0.04		0.54 ± 0.03
DNN		0.64 ± 0.06		0.71 ± 0.07		0.70 ± 0.05
RF		0.42 ± 0.01		0.43 ± 0.04		0.42 ± 0.02

**Table 3:** A list of top substructure fingerprints and their descriptions.

Fingerprints	Description
SubFPC1	Primary Carbon
SubFPC2	Secondary Carbon
SubFPC3	Tertiary Carbon
SubFPC4	Quaternary Carbon
SubFPC5	Alkene
SubFPC12	Alcohol
SubFPC16	Dialkylether
SubFPC18	Alkylarylether
SubFPC26	Tertiary Aliphalitic Amine
SubFPC28	Primary Aromatic Amine
SubFPC32	Secondary Mixed Amine
SubFPC33	Tertiary Mixed Amine
SubFPC88	Carboxylic Acid derivative
SubFPC99	Primary Amide
SubFPC100	Secondary Amide
SubFPC101	Tertiary Amide
SubFPC133	Nitrile
SubFPC137	Vinylogous Ester
SubFPC143	Carbonic Acid Derivatives
SubFPC171	Arylchloride
SubFPC172	Arylfluoride
SubFPC179	Hetero N basic H
SubFPC180	Hetero N basic no H
SubFPC184	Heteroaromatic
SubFPC200	Sulfon
SubFPC214	Sulfonic Derivative
SubFPC279	Annelated Rings
SubFPC287	Spiro
SubFPC294	Trifluoromethyl
SubFPC295	C ONS Bond
SubFPC301	1,5-Tautomerizable
SubFPC302	Rotatable Bond
SubFPC307	Chiral Center Specified

**Table 4:** Summary of the mean and standard deviation of pIC${}_{50}$, MW and AlogP along with their respective chemical clusters.

Cluster No.	pIC${}_{50}$	N	MW	AlogP
1	7.30 ± 1.12	876	456.11 ± 75.65	3.87 ± 1.16
2	7.57 ± 0.68	491	432.38 ± 58.91	3.59 ± 0.95
3	7.70 ± 0.81	137	455.01 ± 43.35	1.68 ± 0.99
4	6.98 ± 0.52	23	333.59 ± 55.50	2.01 ± 1.01
5	9.76 ± 0.75	58	385.45 ± 33.55	1.11 ± 0.69
6	10.04 ± 0.32	38	461.42 ± 44.55	0.74 ± 0.98
7	6.48 ± 0.41	25	436.48 ± 34.56	2.33 ± 0.95
8	8.12 ± 1.09	25	441.67 ± 24.85	3.78 ± 0.50
9	6.06 ± 0.85	20	287.99 ± 30.95	1.30 ± 1.59
10	6.97 ± 0.44	24	283.09 ± 15.28	1.68 ± 0.48

## Supplementary Materials

The following are available online at https://www.mdpi.com/1420-3049/24/23/4393/s1, Table S1: Compounds falling outside the applicability domain of the model as deduced from the Williams plot.

## Abbreviations

The following abbreviations are used in this manuscript:

JAK2	Janus Kinase 2
QSAR	Quantitative Structure-Activity Relationship
DT	Decision Tree
SVM	Support Vector Machine
DNN	Deep Neural Network
RF	Random Forest
RMSE	Root Mean Square Error
CV	Cross Validation
OECD	Organisation for Economic Cooperation and Development
MW	Molecular Weight
ALogP	Octanol-Water Partition Coefficient
nHBDon	Number of Hydrogen Bond Donors
nHBAcc	Number of Hydrogen Bond Acceptors
