# Supplementary material for: Construction of Quantitative Structure Activity Relationship (QSAR) Models to Predict Potency of Structurally Diversed Janus Kinase 2 Inhibitors
Source: Molecules. 2019 Dec 1;24(23):4393. doi: 10.3390/molecules24234393 (PMC6930640; doi:10.3390/molecules24234393)
Supplement: Supplementary file 1 [file molecules-24-04393-s001.pdf]

Table S1. Compounds falling outside the applicability domain of the model as deduced from the Williams plot.

ChEMBL ID	Set	Structure
CHEMBL1923570	Test	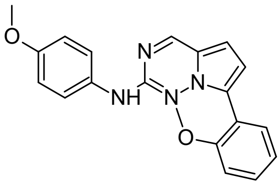
CHEMBL3665202	Test	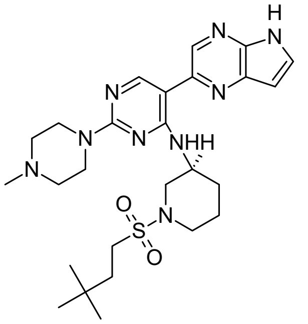
CHEMBL1822511	Test	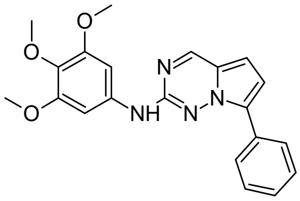
CHEMBL3665178	Test	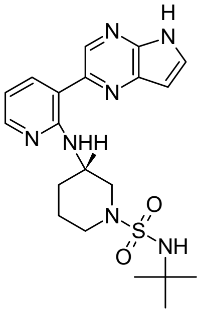

ChEMBL ID	Set	Structure
CHEMBL3652406	Training	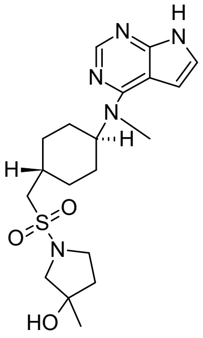
CHEMBL3652404	Training	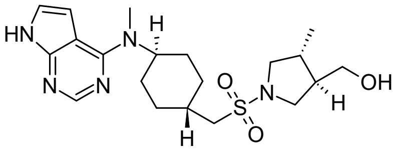
CHEMBL3652408	Training	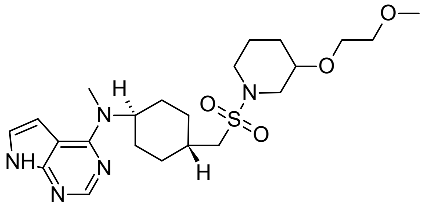
CHEMBL3645094	Test	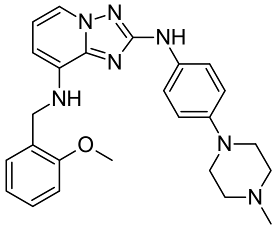
CHEMBL3652402	Test	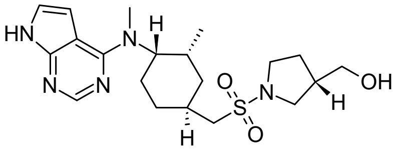

ChEMBL ID	Set	Structure
CHEMBL3652407	Training	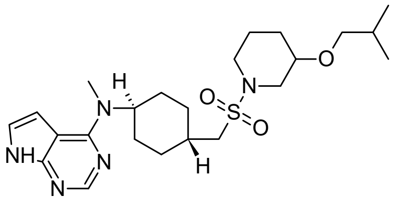
CHEMBL1774053	Test	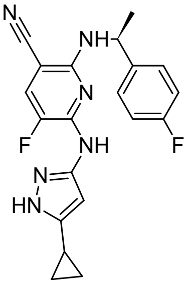
CHEMBL3647751	Test	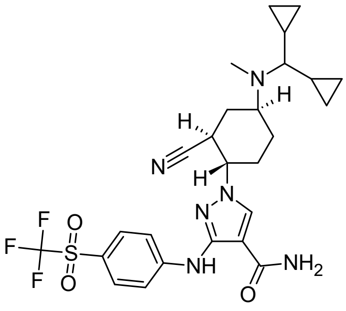
CHEMBL3652409	Test	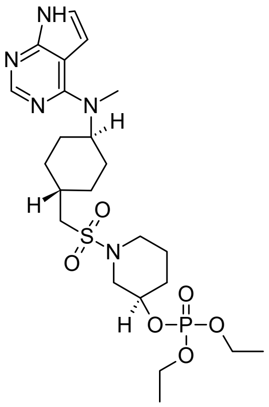

ChEMBL ID	Set	Structure
CHEMBL3652410	Training	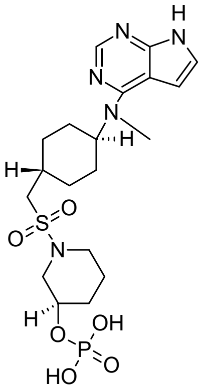
CHEMBL3642321	Training	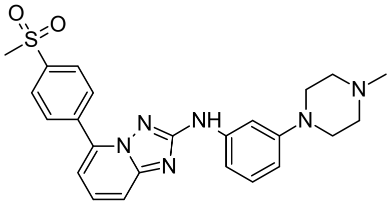
CHEMBL3642293	Test	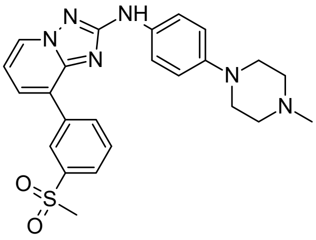
CHEMBL3642275	Test	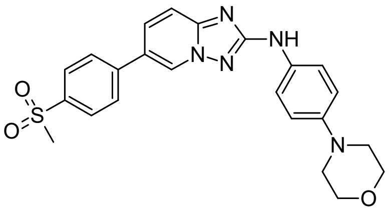

ChEMBL ID	Set	Structure
CHEMBL462168	Training	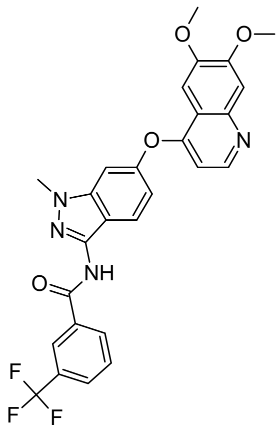
CHEMBL3134612	Test	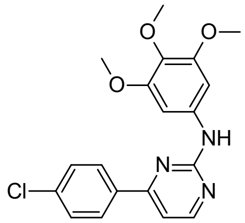
CHEMBL1537091	Test	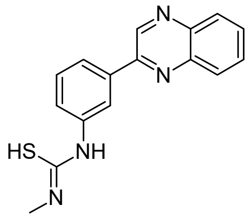
CHEMBL1934349	Test	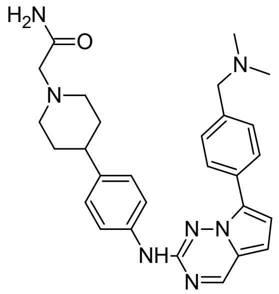

ChEMBL ID	Set	Structure
CHEMBL3645543	Test	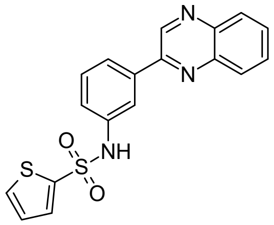
CHEMBL3658866	Test	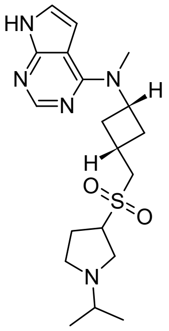
CHEMBL185140	Test	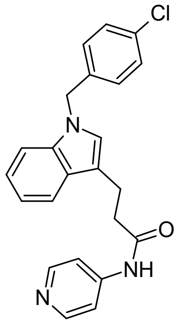
CHEMBL519279	Test	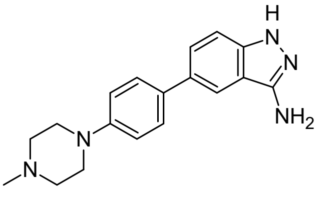

ChEMBL ID	Set	Structure
CHEMBL3665169	Training	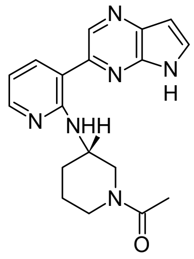
CHEMBL1835029	Test	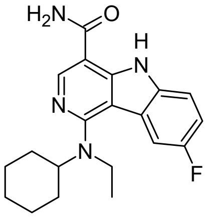
CHEMBL3642251	Test	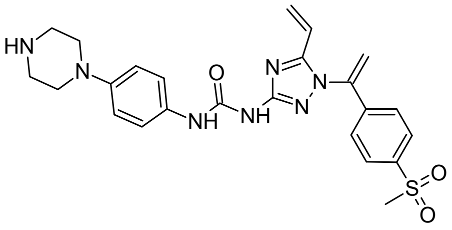
CHEMBL3330106	Test	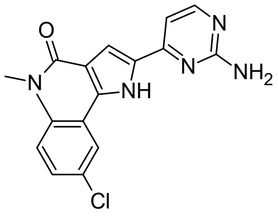
CHEMBL1081378	Test	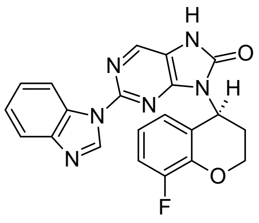

ChEMBL ID	Set	Structure
CHEMBL3622958	Test	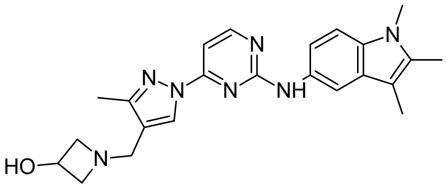
CHEMBL1923591	Training	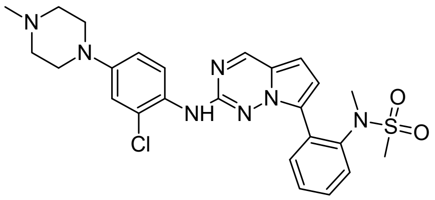
CHEMBL446576	Training	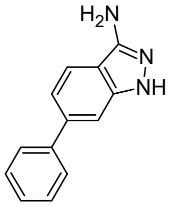
